# Genome-editing Technologies for Gene and Cell Therapy

**DOI:** 10.1038/mt.2016.10

**Published:** 2016-02-16

**Authors:** Morgan L Maeder, Charles A Gersbach

**Affiliations:** 1 Editas Medicine, Cambridge, Massachusetts, USA; 2 Department of Biomedical Engineering, Duke University, Durham, North Carolina, USA; 3 Center for Genomic and Computational Biology, Duke University, Durham, North Carolina, USA; 4 Department of Orthopaedic Surgery, Duke University Medical Center, Durham, North Carolina, USA

## Abstract

Gene therapy has historically been defined as the addition of new genes to human cells. However, the recent advent of genome-editing technologies has enabled a new paradigm in which the sequence of the human genome can be precisely manipulated to achieve a therapeutic effect. This includes the correction of mutations that cause disease, the addition of therapeutic genes to specific sites in the genome, and the removal of deleterious genes or genome sequences. This review presents the mechanisms of different genome-editing strategies and describes each of the common nuclease-based platforms, including zinc finger nucleases, transcription activator-like effector nucleases (TALENs), meganucleases, and the CRISPR/Cas9 system. We then summarize the progress made in applying genome editing to various areas of gene and cell therapy, including antiviral strategies, immunotherapies, and the treatment of monogenic hereditary disorders. The current challenges and future prospects for genome editing as a transformative technology for gene and cell therapy are also discussed.

The realization of the genetic basis of hereditary disease led to the early concept of gene therapy in which “exogenous ‘good' DNA be used to replace the defective DNA in those who suffer from genetic defects”.^1^ More than 40 years of research since this proposal of gene therapy has shown the simple idea of gene replacement to be much more challenging and technically complex to implement both safely and effectively than originally appreciated. Many of these challenges centered on fundamental limitations in the ability to precisely control how genetic material was introduced to cells. Nevertheless, the technologies for addition of exogenous genes have made remarkable progress during this time and are now showing promising clinical results across a range of strategies and medical indications.^2^ However, several challenges still remain. Integrating therapeutic genes into the genome for stable maintenance in replicating cells can have unpredictable effects on gene expression and unintended effects on neighboring genes.^3^ Moreover, some therapeutic genes are too large to be readily transferred by available delivery vectors. Finally, the addition of exogenous genes cannot always directly address dominant mutations or remove unwanted genetic material such as viral genomes or receptors. To address these fundamental limitations of conventional methods for gene addition, the field of gene editing has emerged to make precise, targeted modifications to genome sequences. Here we review the recent exciting developments in the ease of use, specificity, and delivery of gene-editing technologies and their application to treating a wide variety of diseases and disorders.

## Mechanisms of Gene Editing

**Figure 1:**
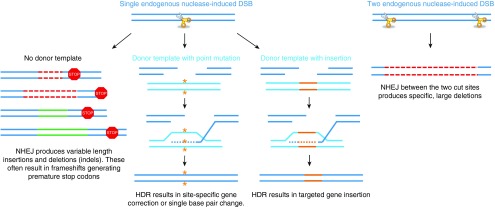
**Mechanisms of double-strand break repair.**

Foundational to the field of gene editing was the discovery that targeted DNA double strand breaks (DSBs) could be used to stimulate the endogenous cellular repair machinery. Breaks in the DNA are typically repaired through one of two major pathways—homology-directed repair (HDR) or nonhomologous end-joining (NHEJ) (**Figure 1**).^4^ HDR relies on strand invasion of the broken end into a homologous sequence and subsequent repair of the break in a template-dependent manner.^5^ Seminal work from the lab of Maria Jasin demonstrated that the efficiency of gene targeting through homologous recombination in mammalian cells could be stimulated by several orders of magnitude by introducing a DSB at the target site.^6,7,8^ Alternatively, NHEJ functions to repair DSBs without a template through direct religation of the cleaved ends.^9^ This repair pathway is error-prone and often results in insertions and/or deletions (indels) at the site of the break. Stimulation of NHEJ by site-specific DSBs has been used to disrupt target genes in a wide variety of cell types and organisms by taking advantage of these indels to shift the reading frame of a gene.^10,11,12,13,14^ Armed with the ability to harness the cell's endogenous DNA repair machinery, it is now possible to engineer a wide variety of genomic alterations in a site-specific manner.

### Gene knockout/mutation

This simplest form of gene editing utilizes the error-prone nature of NHEJ to introduce small indels at the target site. Classical NHEJ directly religates unprocessed DNA ends whereas alternative-NHEJ (also known as microhomology-mediated end joining, or MMEJ) requires end-resection followed by annealing of short single-stranded regions of microhomology and subsequent DNA end ligation.^15^ Active during all stages of the cell cycle, both of these NHEJ pathways repair DNA with a high frequency of mutagenesis resulting in the formation of indels at the site of the break.^15,16^

When the nuclease target site is placed in the coding region of a gene, the resulting indels will often cause frameshifts. In diseases such as Duchenne muscular dystrophy (DMD), where gene deletions result in frameshifts and subsequent loss of protein function, targeted NHEJ-induced indels can be used to restore the correct reading frame of the gene.^17^ However, the most common application of targeted mutagenesis involves inducing frameshift mutations for the purpose of gene knockout. In contrast to traditional gene therapy, which is limited to the addition of exogenous sequence into the genome, the ability to knockout endogenous genes opens a new avenue of therapeutic treatment in which gene function can be permanently disrupted. One application of this approach is to target dominant gain-of-function mutations, such as those found in Huntington's disease. This disease is caused by a repeat expansion on one allele of the huntingtin (*HTT*) gene, leading to the production of a toxic mutant HTT protein. Eliminating this mutant allele by NHEJ-based gene editing could provide clinical benefit to Huntington's patients.^18,19^ In other diseases, it may sometimes be therapeutic to remove the normal function of a gene. The most prominent example of this is the gene-editing approach currently in clinical trials for the treatment of HIV, in which knockout of *CCR5*, the major HIV coreceptor, prohibits viral infection of modified T cells.^20,21,22^ Finally, rather than directly targeting the human genome, knockout of critical genes in invading bacteria or DNA-based viruses could serve as effective anti-microbial treatments.^23,24^

### Gene deletion

In addition to the relatively minor indels resulting from NHEJ, it is possible to delete large segments of DNA by flanking the sequence with two DSBs. Indeed, it has been shown that simultaneous introduction of two targeted breaks can give rise to genomic deletions up to several megabases in size.^25,26,27,28,29^ This approach is useful for therapeutic strategies that may require the removal of an entire genomic element, such as an enhancer region, as has been proposed for the treatment of hemoglobinopathies by deletion of the BCL11A erythroid-specific enhancer region.^30,31^ Additionally, in diseases such as DMD where different internal gene deletions can shift the gene out of frame, the intentional deletion of one or more exons can correct the reading frame and restore the expression of truncated, but partially functional, protein.^32,33,34,35,36,37^

### Gene correction

As opposed to the unpredictable mutations resulting from NHEJ, targeted DSBs can induce precise gene editing by stimulating HDR with an exogenously supplied donor template. Active mainly during the S and G2 phases of the cell cycle, HDR naturally utilizes the sister chromatid as a template for DNA repair.^15,16,38^ However, an exogenously supplied donor sequence may also be used as a repair template.^39^ Thus the codelivery of targeted nucleases along with a targeting vector containing DNA homologous to the break site enables high-efficiency HDR-based gene editing.^6,7,8^ Any sequence differences present in the donor template can thus be incorporated into the endogenous locus to correct disease-causing mutations, as has been demonstrated in many proof-of-concept studies.^40,41,42,43,44,45,46,47,48,49,50^ While plasmids have traditionally been the most commonly used source of donor DNA, recent studies have shown that single stranded oligonucleotides (ssODNs), with as little as 80 base pairs of homology, can serve as efficient donor templates for HDR.^51,52,53^ For cells that are difficult to transfect, viral vectors such as integrase-deficient lentivirus or adeno-associated virus (AAV) can also be used as a source of donor DNA.^54,55,56,57^ In fact, the naturally recombinogenic nature of AAV, especially when combined with the particularly efficient hybrid serotypes such as AAV-DJ, makes them attractive vectors for delivery of the donor template.^54,56,58,59,60,61,62^

### Gene insertion

Although traditional gene therapy has successfully used viral vectors to introduce exogenous genes into the genome, the inability to control the integration site of these viruses raises serious concerns of insertional mutagenesis, as was underscored in the early clinical trials that used murine retroviral vectors.^63,64,65^ The use of a donor template, in which the desired genetic insert is flanked by homology arms including sequences identical to the nuclease cut site, enables site-specific DNA insertion through DSB-induced HDR.^66^ Targeted insertion of therapeutic transgenes into predetermined sites in the genome, such as “safe harbor” loci, alleviates risks of insertional mutagenesis and enables high levels of ubiquitous gene expression.^67,68,69^ To maintain control of gene expression by natural regulatory elements, a wild type copy of the disease-causing gene may be inserted into the corresponding endogenous locus and thus be under the control of its own promoter.^70,71^ An alternative mechanism for targeted transgene insertion is to use nuclease-induced DSBs to create compatible overhangs on the donor DNA and the endogenous site, leading to NHEJ-mediated ligation of the insert DNA sequence directly into the target locus.^72^

## Targeted Nucleases

**Figure 2:**
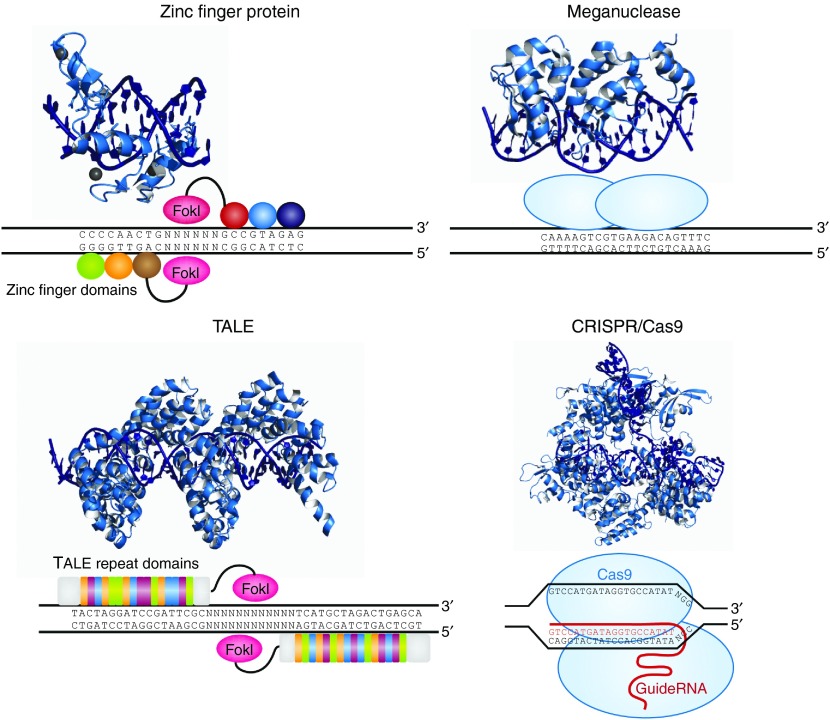
**Common DNA targeting platforms for genome editing.**

Because DSB-induced gene editing relies on the endogenous repair mechanisms of the cell, it is universally applicable to any cell type or organism that employs these methods for DNA repair. The critical element for implementing any of these gene-editing methods is the precise introduction of a targeted DSB. Four major platforms currently exist for inducing these site-specific DSBs: zinc finger nucleases (ZFNs), transcription activator-like effector (TALE)-nucleases (TALENs), meganucleases, and most recently the CRISPR/Cas system (**Figure 2**).

### Zinc finger nucleases

Zinc finger (ZF) proteins are the most abundant class of transcription factors and the Cys_2_-His_2_ zinc finger domain is one of the most common DNA-binding domains encoded in the human genome.^73^ The crystal structure of Zif268 has served as the basis for understanding DNA recognition by zinc fingers.^74,75,76^ In the presence of a zinc atom, the zinc finger domain forms a compact ββα structure with the α-helical portion of each finger making contact with 3 or 4 bp in the major groove of the DNA.^74,77,78^ Tandem fingers in a zinc finger array wrap around the DNA to bind extended target sequences such that a three-finger protein binds a 9 bp target site.

The modular structure of Zif268 suggested that these proteins might provide an attractive framework for engineering novel DNA-binding motifs.^79^ Initial attempts to design ZFs with unique specificities based on a simple set of rules had some success^80,81^; however, combinatorial libraries combined with selection-based methods proved to be a more robust approach for generating individual fingers with novel DNA-binding specificities.^82,83,84,85,86,87^ Following this success, the field was faced with the challenge of engineering multi-finger arrays with novel target sites long enough to be unique in a complex genome. The “modular assembly” approach relies on collections of single-finger modules, either identified in naturally occurring proteins^88^ or selected to bind specific three base pair target sites,^89,90,91,92^ which are then linked in tandem to generate novel proteins.^93,94,95,96,97^ Alternatively, selection-based methods, such as OPEN, may be used to select new proteins from randomized libraries.^98^ While significantly more labor intensive, this method takes into account context-dependent interactions between neighboring fingers within a multi-finger array.^76,99,100,101^ Several methods, including those used by Sangamo Biosciences and the Sigma-Aldrich CompoZr platform, combine these two approaches to assemble novel arrays using archives of multi-finger units that have been preselected to work well together.^13,102,103,104^

The zinc finger nuclease (ZFN) technology was made possible by the discovery that the DNA-binding domain and the cleavage domain of the FokI restriction endonuclease function independently of each other.^105^ By replacing the FokI DNA-binding domain with a zinc finger domain, it is possible to generate chimeric nucleases with novel binding specificities.^106,107^ Because the FokI nuclease functions as a dimer, two ZFNs binding opposite strands of DNA are required for induction of a DSB.^108^ Initial experiments showed that ZFN-induced DSBs could be used to modify the genome through either NHEJ or HDR^10,109,110^ and this technology has subsequently been used to successfully modify genes in human somatic^40,66,98^ and pluripotent stem cells.^42,44,111,112,113^

### TALENs

The discovery of a simple one-to-one code dictating the DNA-binding specificity of TALE proteins from the plant pathogen *Xanthomonas* again raised the exciting possibility for modular design of novel DNA-binding proteins.^114,115^ Highly conserved 33–35 amino acid TALE repeats each bind a single base pair of DNA with specificity dictated by two hypervariable residues. Crystal structures of TALEs bound to DNA revealed that each repeat forms a two-helix structure connected by a loop which presents the hypervariable residue into the major groove as the protein wraps around the DNA in a superhelical structure.^116,117^ These modular TALE repeats can be linked together to build long arrays with custom DNA-binding specificities.^118,119,120,121,122^

Many platforms exist for engineering TALE arrays. The simplest methods use standard cloning techniques to assemble TALEs from archives of plasmids, each consisting of single TALE repeats.^123,124^ Several medium-throughput methods rely on the Golden Gate cloning system to assemble multiple pieces simultaneously in a single reaction.^120,122,125,126,127,128,129^ The highest-throughput methods utilize solid phase assembly^130,131,132^ or ligation-independent cloning techniques.^133^

Building off the foundation laid by a decade of ZFN-induced genome editing, the discovery of TALEs as a programmable DNA-binding domain was rapidly followed by the engineering of TALENs. Like ZFNs, TALEs were fused to the catalytic domain of the FokI endonuclease and shown to function as dimers to cleave their intended DNA target site.^119,121,134,135^ Also similar to ZFNs, TALENs have been shown to efficiently induce both NHEJ and HDR in human somatic^119,132,134^ and pluripotent stem cells.^53,136^

TALENs can be engineered to target virtually any sequence given that their only targeting restraint is the requirement for a 5' T, specified by the constant N-terminal domain, for each array. This unlimited targeting range, in addition to the ease of engineering new proteins, makes TALENs an attractive platform for targeted gene editing. Conversely, the large size and repetitive nature of TALE arrays presents a hurdle for *in vivo* delivery of these proteins. As opposed to a 30 amino acid zinc finger, which binds three bases of DNA, TALENs require 34 amino acids to specify a single base pair and this size difference can prohibit delivery of both TALEN monomers in a single viral vector with limited packaging capacity. Additionally, the unstable nature of tandem repeats, such as those present in TALENs, makes it challenging to package repetitive sequences in viral systems. Indeed, TALENs delivered by lentivirus have been shown to be susceptible to rearrangements,^137^ although this phenomenon may be mitigated by codon diversification between the repeats.^138^ Adenoviral systems have also been used to successfully deliver TALENs.^139^

### Meganucleases

Meganuclease technology involves re-engineering the DNA-binding specificity of naturally occurring homing endonucleases. The largest class of homing endonucleases is the LAGLIDADG family, which includes the well-characterized and commonly used I-*Cre*I and I-*Sce*I enzymes.^140^ Through a combination of rational design and selection, these homing endonucleases can be re-engineered to target novel sequences.^141,142,143,144,145,146,147,148^ While many studies show promise for the use of meganucleases in genome editing,^149,150,151,152^ the DNA-binding and cleavage domains of homing endonucleases are difficult to separate, and the relative difficulty of engineering proteins with novel specificities has traditionally limited the use of this platform. To address this limitation, chimeric proteins comprising fusions of meganucleases, ZFs, and TALEs have been engineered to generate novel monomeric enzymes that take advantage of the binding affinity of ZFs and TALEs and the cleavage specificity of meganucleases.^153,154,155,156^ One potential advantage associated with meganuclease technology is that DSB-formation by these enzymes results in a 3' overhang, which may be more recombinogenic for HDR than the 5' overhang generated by FokI cleavage. Additionally, meganucleases are the smallest class of engineered nucleases, making them potentially amenable to all standard gene delivery methods. In fact, multiple meganuclease monomers could be readily packaged into single viral vectors to simultaneously create multiple DSBs.

### CRISPR/Cas nucleases

CRISPR-Cas RNA-guided nucleases are derived from an adaptive immune system that evolved in bacteria to defend against invading plasmids and viruses. Decades of work investigating CRISPR systems in various microbial species has elucidated a mechanism by which short sequences of invading nucleic acids are incorporated into CRISPR loci.^157^ They are then transcribed and processed into CRISPR RNAs (crRNAs) which, together with a trans-activating crRNAs (tracrRNAs), complex with CRISPR-associated (Cas) proteins to dictate specificity of DNA cleavage by Cas nucleases through Watson-Crick base pairing between nucleic acids.^158,159,160,161^ Building off of two studies showing that the three components required for the type II CRISPR nuclease system are the Cas9 protein, the mature crRNA and the tracrRNA,^162,163^ Doudna, Charpentier and colleagues showed through *in vitro* DNA cleavage experiments that this system could be reduced to two components by fusion of the crRNA and tracrRNA into a single guide RNA (gRNA). Furthermore, they showed that re-targeting of the Cas9/gRNA complex to new sites could be accomplished by altering the sequence of a short portion of the gRNA.^164^ Thereafter, a series of publications demonstrated that the CRISPR/Cas9 system could be engineered for efficient genetic modification in mammalian cells.^165,166,167,168^ Collectively these studies have propelled the CRISPR/Cas9 technology into the spotlight of the genome-editing field.

The only sequence limitation of the CRISPR/Cas system derives from the necessity of a protospacer-adjacent motif (PAM) located immediately 3' to the target site. The PAM sequence is specific to the species of Cas9. For example, the PAM sequence 5'-NGG-3' is necessary for binding and cleavage of DNA by the commonly used Cas9 from *Streptococcus pyogenes.*^169,170,171^ However, Cas9 variants with novel PAMs may be engineered by directed evolution, thus dramatically expanding the number of potential target sequences.^172,173^ Cas9 complexed with the crRNA and tracrRNA undergoes a conformational change and associates with PAM motifs throughout the genome interrogating the sequence directly upstream to determine sequence complementarity with the gRNA.^171,174,175,176,177^ The formation of a DNA-RNA heteroduplex at a matched target site allows for cleavage of the target DNA by the Cas9-RNA complex.^171^

Unlike the three nuclease systems discussed above, CRISPR/Cas nucleases do not require the engineering of novel proteins for each DNA target site. The relative ease with which new sites can be targeted, simply by altering the short region of the gRNA that dictates specificity, makes this system a highly attractive method for introducing site-specific DSBs. Additionally, because the Cas9 protein is not directly coupled to the gRNA, this system is highly amenable to multiplexing through the concurrent use of multiple gRNAs to induce DSBs at several loci. Because the rich diversity of natural CRISPR systems has been largely understudied, it is reasonable to expect many new CRISPR-based gene-editing technologies to emerge, including non-Cas9 based type II systems such as the recently described RNA-guided endonuclease Cpf1 and others.^178,179^

### Specificity of targeted nucleases

The efficacy of targeted gene editing relies on cleaving the DNA in a site-specific manner while mitigating, or ideally preventing, collateral damage to the rest of the genome. For this reason, the specificity of targeted nucleases is a major focus of the gene-editing field. Modifications to the FokI dimerization domain dramatically increased the specificity of ZFNs and TALENs by requiring two obligate heterodimers to bind the target DNA in a specific orientation and spacing.^180,181,182,183^ Reminiscent of the architecture of ZFNs and TALENs, the inactivation of Cas9 nuclease domains to create Cas9 nickases or Cas9-FokI fusions has increased specificity by requiring two gRNA/Cas9 complexes, each cleaving a single strand of DNA, to come together at a precise distance and orientation in order to generate a DSB.^184,185,186,187^ Additionally, reducing the length of complementarity between the gRNA and the target site from 20 to 17 nucleotides increases the specificity of DNA cleavage by Cas9 from *S. pyogenes*.^188^ Recently, structure-guided protein engineering has been used to develop novel Cas9 variants with increased specificity properties.^189,190^ These improvements have significantly alleviated initial concerns over the specificity of CRISPR/Cas nucleases.^191,192,193^ However, regardless of the nuclease technology, it is difficult to determine the full spectrum of off-target cleavage in a complex genome. Until recently, specificity studies were largely limited to *a priori*, *in silico* identification of potential off-target sites that could be informed by surrogate assays with purified proteins or viral integrations at double-strand breaks.^194,195,196^ Whole-genome sequencing of a small number of clones derived from single cells has verified the lack of off-target effects in these select populations, but cannot identify sites that are cleaved at low frequencies in bulk cell populations.^197,198,199^ Interrogation of DNA-binding specificity by ChIP-seq was greatly informative for understanding target site recognition, but the vast majority of the off-target binding sites were not predictive of nuclease activity.^200,201,202^ Recent development of methods for unbiased, genome-wide assays to determine specificity have significantly advanced the ability to characterize nuclease specificity with a degree of sensitivity that was not previously possible.^195,203,204,205,206^ These new methods will likely be critical to advancing targeted gene-editing nucleases as therapeutics.

## Delivery of Genome-Editing Tools

**Figure 3:**
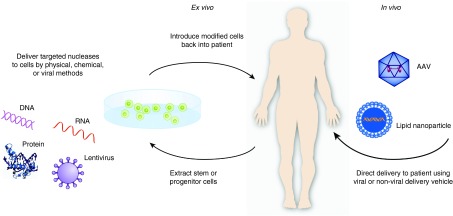
***Ex vivo* and *in vivo* strategies for therapeutic genome editing.**

Efficient and safe delivery to target cells and tissues has been the long-standing challenge to successful gene therapy strategies (**Figure 3**). This challenge extends to genome-editing methods as well, where the nucleases, and in the case of the CRISPR/Cas9 system, a gRNA, must be efficiently delivered. Moreover, the dose of the donor template DNA is important to ensuring efficient homologous recombination. The duration and magnitude of nuclease expression is a critical parameter for the level of both on-target and off-target nuclease activity. Maximizing the efficiency of delivery is particularly important since gene editing is an inherently stochastic event occurring in only a fraction of the cells in which the nuclease is expressed.

The most widely reported method for introducing nucleases into cells in proof-of-principle studies is transfection of plasmid DNA carrying nuclease and gRNA expression cassettes. Although simple and straightforward, this method is not ideal for most gene and cell therapies due to low efficiency of transfection of primary cells, DNA-related cytotoxicity, the presence of bacterial DNA sequences in plasmid backbones, and the possibility of random integration of plasmid fragments into the genome. Consequently, electroporation of mRNA encoding the nucleases and gRNAs generated through *in vitro* transcription has become a preferred method for *ex vivo* gene editing of primary cells relevant to gene therapy, such as T cells and hematopoietic stem cells (HSCs).^168,207^ Alternatively, the direct delivery of purified nuclease proteins or Cas9 protein-gRNA complexes has also been very successful in achieving high levels of gene editing, either by electroporation^208,209^ or fusion to cell-penetrating peptides, which obviates electroporation-mediated toxicity.^210,211,212^ Chemical modification of the gRNAs can further increase the robustness of gene editing in primary cells by increasing stability and/or decreasing innate immune responses.^207^ These studies have collectively shown that by restricting the duration of nuclease activity with short-lived mRNA or proteins, off-target effects can be minimized compared to plasmid-based delivery. Future efforts will likely take advantage of emerging nanoparticle formulations for efficient and nontoxic delivery.^213^

For many applications, viral vectors are still the optimal vehicle to maximize the efficiency of delivery while minimizing cytotoxicity.^214^ In particular, lentiviral vectors have been optimized for highly efficient transduction of T cells and HSCs; however these vectors also integrate into the genome and stably express their transgene cargo. In order to take advantage of the efficiency of lentiviral transduction while limiting the duration of nuclease expression in target cells, integrase-deficient lentiviral vectors have been used to transiently deliver genome-editing tools to target cells.^57,111^ Similarly, adenoviral systems can also achieve high levels of transduction of a variety of cell types *ex vivo* while providing only transient nuclease expression.^20,137,139^ Both lentiviral and adenoviral vectors also have the advantage of sufficient packaging capacity to carry multiple nucleases or gRNA expression cassettes for multiplex editing of several loci.^215^

*In vivo* gene editing presents additional challenges of tissue-specific targeting, distribution of the vector, and immunogenicity and biocompatibility of the carrier. Although several examples of plasmid delivery to the liver have shown important proof-of-principle of *in vivo* gene editing in animal models,^216,217,218^ translating these strategies to human therapy is not yet feasible. However, *in vivo* gene delivery with AAV to the liver, eye, nervous system, and skeletal and cardiac muscle has shown impressive efficacy in both preclinical models and clinical trials.^219^ Consequently, AAV is also a promising system for delivery of gene-editing nucleases to target tissues.^220^ Furthermore, the natural recombinogenic properties of AAV make it a desirable vector for delivery of DNA repair templates.^56,61,62,221,222,223,224^ Although some studies have shown targeted recombination of genomic loci with AAV vectors in the absence of nucleases,^58,71^ the efficiencies are significantly lower than reports that include nucleases. Further studies are required to understand which disease indications can be robustly addressed at lower efficiencies of gene editing.

Although AAV has shown considerable promise for *in vivo* gene delivery, its packaging capacity is limited to less than ~4.8 kb of DNA. This has posed a challenge for the delivery of large nucleases such as TALENs, that require two monomers each encoded by cDNAs greater than four kb in size, and the commonly used *S. pyogenes* Cas9 nuclease that is encoded by a ~4.2 kb cDNA. Trans-splicing vectors have been designed to recombine within cells to expand the size of transgenes delivered by AAV,^225^ but the efficiency of expression is significantly lower than genes delivered by a single AAV. A number of smaller Cas9 orthologs exist, and the ~3.1 kb Cas9 from *S. aureus* has been thoroughly characterized and shown to mediate highly efficient gene editing *in vivo* following AAV delivery.^35,36,226,227^ This important advance is critical to enabling facile and robust *in vivo* gene editing with the CRISPR/Cas9 system. It is particularly advantageous for developing a translatable gene therapy product that can be packaged in a single vector.

## Gene Therapy Applications

**Figure 4:**
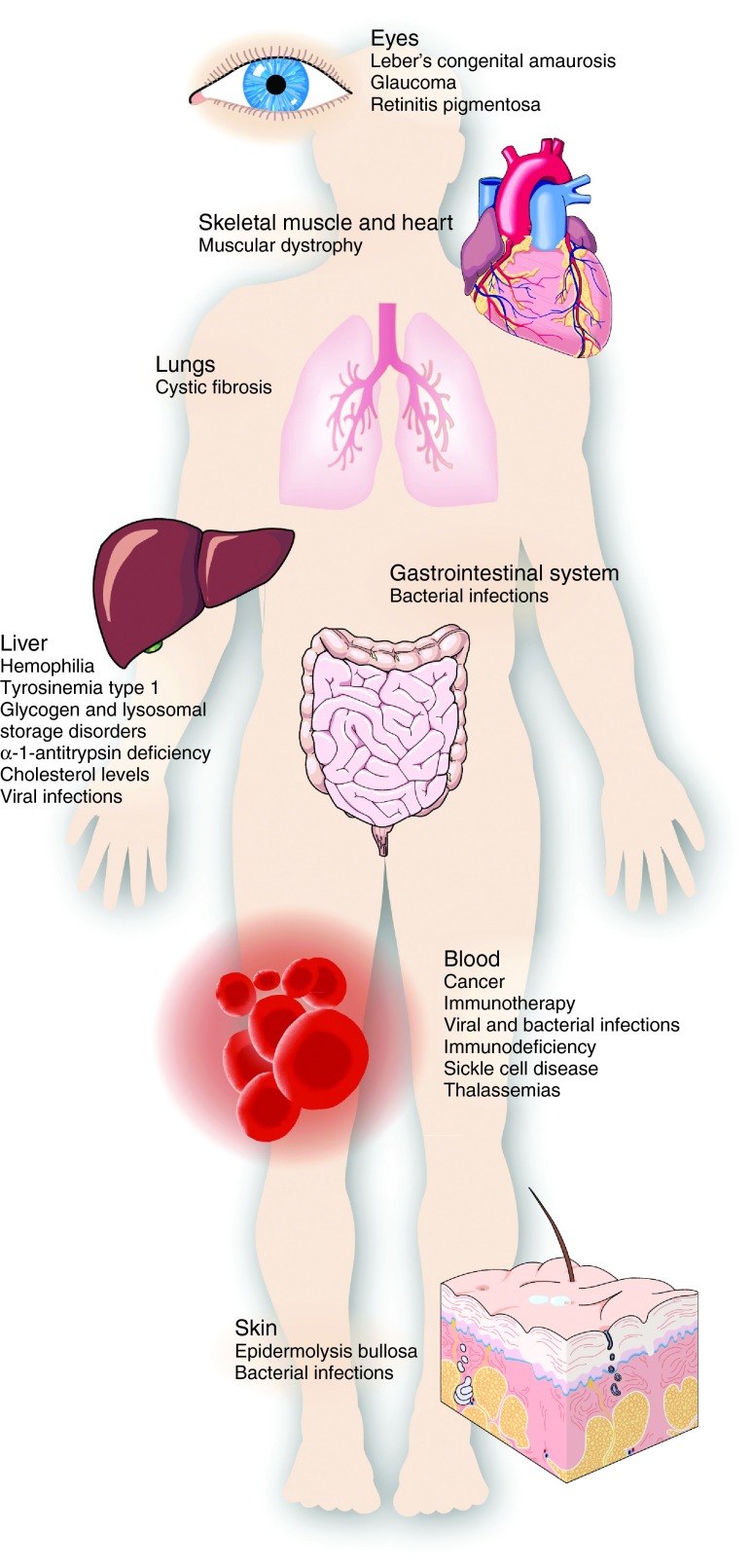
**Diversity of targets for therapeutic genome editing.**

The ability to manipulate any genomic sequence by gene editing has created diverse opportunities to treating many different diseases and disorders (**Figure 4**). Here, we discuss the major categories of disease indications that have been pursued in preclinical models (**Table 1**), as well as highlight the ongoing or planned clinical trials using gene-editing strategies (**Table 2**).

### Antiviral strategies

The most straightforward application of gene editing is to use the relatively efficient NHEJ mechanism to knockout genes in an *ex vivo* autologous cell therapy, where somatic cells can be isolated, modified, and delivered back to the patient. Moreover, one of the most compelling applications of gene editing is the prevention of viral infection or replication. Thus the most advanced gene-editing strategy to date is the *ex vivo* modification of T cells to knock out the *CCR5* coreceptor used for primary HIV infection.^20^ This early study demonstrated decreased viral loads and increased CD4+ T-cell counts in HIV-infected mice engrafted with T cells in which the *CCR5* gene had been knocked out by zinc finger nucleases.^20^ This was later followed by demonstration of similar results following gene editing and transplantation of CD34+ HSCs into irradiated mice, allowing for protection of all blood cell lineages from CCR5-tropic HIV infection.^21,228^ These studies have led to a series of clinical trials (**Table 2**) evaluating this approach in HIV-positive human patients. Thus far the studies show safe engraftment and survival of *CCR5*-modified T cells and control of viral load in some patients, providing promising proof-of-principle of a gene-editing approach in humans.^22^ Interestingly, data from this study showed a greater clinical efficacy in a patient that was already heterozygous for the naturally-occurring ▵32 mutation, suggesting that gene-editing efficiency may be a critical factor for success.

Building on these promising studies with ZFNs, several other efforts have developed similar gene-editing strategies to knockout *CCR5* with TALENs,^134,229^ CRISPR/Cas9 (refs. 229, 230) and meganucleases.^231^ Other work has expanded beyond targeting only *CCR5* to enhance resistance to HIV infection. This includes targeting the CXCR4 coreceptor^232^ or *PSIP1* gene encoding the LEDGF/p75 protein required for HIV integration.^233,234^ Some studies have used targeted gene integration into the *CCR5* gene by HDR to simultaneously knockout *CCR5* and introduce anti-HIV factors.^235^ Finally, complete excision of the HIV genome from infected cells using nucleases that target sequences in the long terminal repeats (LTRs) flanking the viral genome has also been reported.^236^ Thus, a variety of next-generation gene-editing strategies for preventing HIV infection and replication are on the horizon.

Beyond addressing HIV infection, all of the gene-editing platforms have also been applied to various other viral pathogens^23^ including hepatitis B virus,^217,218,237,238,239,240,241,242^ herpes simplex virus,^243,244,245^ and human papilloma virus.^246^ These strategies typically involve removing viral genomes by degradation following nuclease cleavage and by targeting genes essential for genome stability, maintenance, and replication. While many of these early studies focused on proof-of-principle reduction of viral load in cell culture or following hydrodynamic plasmid DNA delivery to mice, recent studies using AAV delivery of gene-editing tools directly to the mouse liver provides a plausible path for scalability and clinical translation.^238^ A general challenge of antiviral therapies is the high mutability of viral targets. This is a compelling argument in favor of targeting host genes, such as CCR5, but may also be addressed by simultaneous targeting of multiple critical sites in the viral genome.

### Cancer immunotherapy

Cancer immunotherapy has been widely recognized as one of the greatest advances in biomedical research in recent years.^247^ In particular, adoptive T-cell immunotherapy, in which autologous T cells are engineered to attack cancer antigens *ex vivo* and transferred back to the patient, has been impressively successful at treating some cases of lymphoma, leukemia, and melanoma.^248^ Despite these successes and promising ongoing clinical trials, there are several areas in which T-cell immunotherapy could be potentially improved by gene editing. Here, both the efficacy against diverse tumor types and the ability to manufacture cell products that can be applied to a broad patient population could be enhanced through gene-editing techniques. For example, a promising strategy for immunotherapy involves engineering T cells to express synthetic receptors known as chimeric antigen receptors, or CARs, that recognize epitopes on cancer cells. Such CAR T cells have been particularly successful in treating B-cell lymphoma by targeting the CD19 cell surface antigen.^247,248^ However, one limitation of this approach is that these modified T cells express both the endogenous T-cell receptor as well as the engineered CAR. Because these receptors function as dimers, the natural and engineered receptors can dimerize and interact, resulting in unpredictable epitope specificity and potentially reducing therapeutic potency. To address this limitation, several studies have focused on knocking out the endogenous T-cell receptors with engineered nucleases.^154,249,250,251^

A major challenge to the development of broadly translatable T-cell immunotherapies is the need to use autologous cells to avoid immune rejection. To address this, gene editing has been used to knockout the human leukocyte antigen (HLA) by which the immune system discriminates self and foreign cells.^252^ Importantly, this approach may be broadly useful for allogeneic cell therapy beyond T-cell immunotherapy. For example, similar approaches have been applied in human pluripotent cells potentially having diverse uses in regenerative medicine^252^ as well as in endothelial cells that could be used for allogeneic vascular grafts.^253^

Another major obstacle to successful T-cell immunotherapy is the inhibition of T-cell effector functions by the expression of checkpoint inhibitors on the surface of tumor cells. For example, the binding of such inhibitors to the PD-1 receptor on T cells is well documented to block T-cell effector function and induce apoptosis and exhaustion. PD-1 receptor inhibition thus provides a mechanism by which cancer cells successfully evade the immune system. As a strategy to overcome this, gene editing has been used to knockout PD-1 in T cells,^254^ leading to increased T-cell effector function.^209,254^ The success of this gene-editing strategy is likely extendable to other checkpoint inhibitor pathways that cancer cells exploit to circumvent immunosurveillance, and thus may be a critical technology for broadly enabling immunotherapy for diverse cancer types.

Finally, for indications such as glioblastoma, the apoptosis of the engineered T cells resulting from post-surgery anti-inflammatory glucocorticoid steroid treatment severely limits the efficacy of T-cell immunotherapy. In order to create a glucocorticoid-resistant T-cell source, gene editing was used to knockout the endogenous T-cell receptor.^255^ This led to successful anti-glioma T-cell therapy in mouse models^255^ and was the basis of a subsequent clinical trial (**Table 2**).

### Hematologic disorders

The first gene therapy clinical trials involved the *ex vivo* retroviral delivery of a therapeutic adenosine deaminase (ADA) transgene to T cells to treat children with severe combined immunodeficiency (ADA-SCID),^256^ and later the treatment of X-linked SCID (X-SCID) by retroviral gene delivery to CD34+ hematopoietic stem cells (HSCs).^257^ This early focus on *ex vivo* gene therapy for immunodeficiency was based on the desperate need to develop treatment for these otherwise fatal disorders as well as the availability of methods for efficient retroviral gene delivery to cells in culture. The subsequent observation that this early protocol can lead to insertional mutagenesis was a primary catalyst for the gene-editing field and demonstrated the need for correction of gene mutations in these cells, in contrast to transgene delivery.^63^ In fact, the first example of endogenous gene correction in human cells focused on the IL2 receptor common gamma chain that is mutated in X-SCID,^40^ and this approach was more recently extended to gene correction in CD34+ HSCs.^57^ Gene-editing tools have also been developed to correct gene mutations associated with ADA-SCID^258^ and radiosensitive SCID, caused by impaired DNA-dependent protein kinase (DNA-PK) activity.^259^ Thus, the ability to efficiently alter gene sequences in T cells, CD34+ HSCs, and human pluripotent cells can provide therapeutic gene-editing strategies for a broad range of different human immunodeficiencies.

Similarly, the establishment of gene editing in CD34+ HSCs and human pluripotent cells capable of differentiating into erythroid progenitors has provided new options for treating other hematologic disorders, including sickle cell disease, caused by a specific E6V point mutation in the β-globin gene, and β-thalassemia, caused by other types of mutations to β-globin. These globin mutations have been corrected by gene editing both in human iPSCs that can be differentiated into functional erythrocytes^42,48,260^ and directly in CD34+ HSCs.^261^ Similar approaches have been developed for targeted integration of therapeutic transgenes into safe harbor sites in human iPSCs for α-thalassemia^69^ and Fanconi anemia.^262^

Sickle cell disease and β-thalassemia are unique in that deficiencies in β-globin function or expression can be compensated for by inducing upregulation of γ-globin, which is expressed during fetal development but silenced after birth. BCL11A is a transcriptional regulator that suppresses the expression of γ-globin, and thus the knockout of BCL11A has been proposed as an approach to treat both sickle cell disease and β-thalassemia. However, the absence of BCL11A in all hematopoietic lineages was observed to be detrimental in nonerythroid cells. Interestingly, an enhancer element was discovered that specifically coordinates BCL11A in erythroid cells and inactivation of this enhancer by gene editing leads to suppression of BCL11A and upregulation of γ-globin only in cells of the erythroid lineage.^30,31,263^ Thus, this approach provides both a mechanism of gene-editing therapy for sickle cell disease and β-thalassemia, but more broadly suggests a general strategy of therapeutic modulation of gene expression through the targeted editing of cell type-specific enhancers.

### Liver-targeted gene editing

Beyond the *ex vivo* gene editing of blood and immune cells, there is intense interest in gene editing *in vivo* for gene correction and targeted gene addition to tissues for which cell transplantation is challenging or impractical. This requires the efficient delivery of gene-editing nucleases and donor vectors to target tissues. The first demonstration of highly efficient, nuclease-mediated gene editing *in vivo* used AAV vectors to deliver ZFNs and a factor IX cDNA, without a promoter, to the liver of a mouse model of hemophilia B.^70^ Cleavage of the first intron of a mutated human factor IX gene by ZFNs catalyzed the efficient integration of the factor IX cDNA into the locus, leading to correction of the hemophilic phenotype. This first study was performed in neonates in which the hepatocytes are actively dividing and thus homology-directed repair pathways are active. Notably, a subsequent study demonstrated efficacy in adult mice in which the hepatocytes have presumably exited the cell cycle, although the integrations resulted from a combination of HDR- and NHEJ-mediated events.^264^ Additional studies are necessary to determine the role of cell cycle and gene editing with AAV and other delivery vectors in various tissue types.

Targeted gene correction in the liver has the potential to treat many different diseases, including clotting disorders such as hemophilia A and hemophilia B, as well as lysosomal storage disorders including Fabry disease, Gaucher disease, Pompe disease, von Gierke disease, and Hurler and Hunter syndromes. However, each of these patient populations is relatively small and the types of mutations to each gene involved in these diseases are diverse. Therefore, the cost of clinical development and regulatory approval to develop safe and efficacious gene-editing tools for each of these diseases may be prohibitive. Moreover, it is unclear whether sufficient levels of targeted transgene integration or gene correction could be achieved to reach therapeutic efficacy if driven by the corresponding natural endogenous promoter for each gene. A clever approach to address each of these challenges is the targeted integration of therapeutic genes into the albumin locus downstream of the endogenous albumin promoter.^71,265^ Because albumin is very highly expressed, even low levels of targeted gene integration to this site are likely to lead to therapeutic levels of gene expression. Moreover, this genomic “safe harbor” can be used for diverse diseases, including those listed above, such that a single validated gene-editing reagent can be used for a significantly larger patient population. This approach has been used effectively in mouse models with AAV-based homologous donor templates to treat hemophilia without nucleases^71^ and with ZFNs that are likely to dramatically enhance targeting efficiency.^265^

The advent of the CRISPR/Cas9 system has made *in vivo* gene-editing tools more broadly available to the scientific community and thus many recent studies have used this approach for both developing disease models and strategies for gene therapy. The first example of *in vivo* gene editing with CRISPR/Cas9 involved the correction of a mouse model of hereditary tyrosinemia type I following hydrodynamic tail vein injection of plasmid DNA into mice.^216^ Although overall gene-editing efficiencies were relatively low (~0.4%), this model allows for selection of corrected cells to repopulate the liver and thus it was possible to demonstrate correction of the disease phenotype. Although the method of naked DNA delivery to the liver is likely not translatable to humans, this study was a landmark in demonstrating *in vivo* gene editing with CRISPR/Cas9 in adult tissues.

Beyond gene correction, the disruption of particular genes in the liver may also have a beneficial effect. For example, the *PCSK9 *gene encodes a proteinase that induces degradation of the low density lipoprotein receptor (LDLR). Decreased LDLR levels lead to lower metabolism of LDL cholesterol (LDL-C), increased LDL-C levels, and increased risk for cardiovascular disease. The discovery of natural genetic variation leading to high or low PCSK9 activity and corresponding cholesterol levels has led to intense interest in PCSK9-blocking drugs for lowering cholesterol.^266,267^ In contrast to continuous drug administration, two different studies have shown that a single treatment of Cas9 and a PCSK9-targeted gRNA delivered to the liver can lead to efficient gene knockout and lowered cholesterol levels.^226,268^

Finally, in addition to gene editing in the liver, new methods for culture and differentiation of human pluripotent cells into functional hepatocytes are providing options for *ex vivo* cell correction and engraftment into the liver. For example, α-1-antitrypsin mutations were seamlessly corrected by gene editing in human iPSCs and subsequently differentiated into liver cells that expressed the restored gene.^45^ Although this type of cell-based product may be significantly more complex than a virus-based drug, the strategy described in this study allows for a comprehensive genomic analysis of the modified cells.

### Neuromuscular disorders

Advances in gene delivery and cell transplantation to the central nervous system and skeletal and cardiac muscle have created new opportunities for gene and cell therapy for many neuromuscular disorders, including DMD, the limb girdle muscular dystrophies, spinal muscular atrophy, Friedreich's ataxia, Huntington's disease, and amyotrophic lateral sclerosis (ALS). Amongst this class of diseases, genome editing has thus far advanced most prominently for DMD, although possible strategies to apply genome editing to other conditions could be envisioned. DMD is caused by mutations to the dystrophin gene, most commonly large deletions that shift the downstream gene fragment out of frame and render the protein product nonfunctional. Because the coding sequence of the dystrophin gene is exceptionally large (14 kb), it cannot be packaged into size-restricted viral delivery vectors. Although truncated minigenes have been developed that do fit into viral vectors, they are only partially functional compared to the full-length gene and their ability to reverse the human disease remains to be determined. For these reasons, and because there is currently no available approved therapy for DMD, gene editing to repair the endogenous gene is particularly compelling. Early reports suggested a mechanism to repair the dystrophin gene with gene editing,^269^ which was followed by proof-of-principle experiments in cultured cells from DMD patients demonstrating dystrophin gene repair by targeted integration of the deleted exons^270^ or restoration of dystrophin protein expression by targeted shifting of the reading frame by NHEJ-mediated indels.^17^ However, these two strategies suffer from addressing only a limited patient population with any particular gene-editing strategy or lacking predictable and reliable editing outcomes due to the reliance on stochastic NHEJ-mediated DNA repair, respectively. Therefore, more recent studies have focused on deleting one or more exons with a combination of nucleases to generate precisely restored protein products and address larger fractions of the DMD patient population.^32,33,34^ This includes a single strategy of deleting >300 kb of genomic DNA comprising exons 45–55 that could be applicable to restoring dystrophin expression in 62% of DMD patients.^33^ In order to develop this into an approach that could potentially be applied clinically to DMD patients, recent work has incorporated the CRISPR/Cas9 system into AAV vectors with tropism for skeletal and cardiac muscle.^35,36,37^ When applied locally via intramuscular injection or systemically via intravenous injection to a mouse model of DMD, gene editing by CRISPR/Cas9 restored expression of the dystrophin protein and improved muscle pathology and strength. Notably, one study showed relatively efficient *in vivo* gene editing of Pax7-positive muscle progenitor cells that may act as a renewable source of cells in which the dystrophin gene has been repaired.^36^ This translational approach builds on demonstration of *in vivo* gene editing in skeletal muscle with adenoviral delivery^271^ and correction of dystrophin mutations in single-cell mouse embryos^41^ to reverse disease symptoms. In the future, these efforts may be extended to cell therapies by using patient-derived cell types, such as iPS cells, that could be modified by gene correction or targeted dystrophin transgene insertion^272^ and expanded to large numbers and efficiently engrafted into muscle tissue.^34,273^

### Skin disorders

The development of engineered skin grafts from autologous and allogeneic cells, including iPS cells, is creating new opportunities for treating genetic diseases that affect the skin. For example, recessive dystrophic epidermolysis bullosa is a disease caused by mutations to the gene encoding type VII collagen. This disruption of type VII collagen expression results in extensive skin blistering. This may be treatable by correcting patient cells with genome editing and using those cells to engineer autologous skin grafts.^274^ In one study, the mutations to the type VII collagen gene were corrected in primary patient fibroblasts that were then reprogrammed to iPS cells which could be used to form skin structures *in vivo*.^275^ Another study also corrected the disease-causing mutation in patient iPS cells, and used these cells to generate epithelial keratinocyte sheets, resulting in stratified epidermis *in vitro* in organotypic cultures and *in vivo* in mice.^276^

### Ocular disorders

Recent successes in clinical trials for the treatment of Leber Congenital Amaurosis type 2 (LCA2) have propelled retinal disorders into the spotlight of the gene therapy field. Using a gene augmentation approach, subretinal injection of AAV encoding the full *RPE65* gene was found to be both safe and efficacious in several concurrent trials.^277,278,279,280^ LCA is the leading cause of childhood blindness and is caused by mutations in at least 18 different genes.^281^ LCA10, the most common form of LCA, is caused by mutations in the approximately 7.5kb *CEP290* gene, and is therefore not amenable to the standard gene therapy approach employed for LCA2 due to the large size of the disease-causing gene. While an *in vitro* proof-of-concept study used lentivirus to deliver the full transgene to iPSC-derived photoreceptor precursor cells,^282^ the proven safety of subretinal AAV delivery makes a gene-editing strategy, in which the nuclease components are delivered via AAV, particularly attractive. As proof-of-principle of gene editing for this disease, *S. aureus* Cas9 was used to delete an intronic region in the *CEP290* gene containing a frequent mutation that creates an aberrant splice site which disrupts the gene coding sequence.^283^ Deletion of this intronic region restored proper CEP290 expression. Gene editing is also uniquely positioned to address autosomal dominant disorders, such as forms of primary open angle glaucoma and retinitis pigmentosa, which could potentially be treated by targeted knockout of the *MYOC* and *RHO* genes, respectively.

### Respiratory disorders

Cystic fibrosis is caused by mutations to the CFTR chloride channel. Loss of function of this chloride channel results in dysregulation of epithelial fluid transport in several organs. In particular, loss of proper fluid transport in the lung results in thickening of the mucus and thus frequent infection and complications breathing. Gene editing has been used to repair the CFTR mutations in cultured patient intestinal stem cells^50^ and iPS cells that could be subsequently differentiated into epithelial cells.^284,285^ Although a long-standing challenge for gene therapy and gene editing for cystic fibrosis has been achieving efficient gene delivery to the lung epithelium, a recent study demonstrating functional correction of mice with cystic fibrosis following intranasal delivery of nanoparticles carrying triplex-forming peptide nucleic acid molecules is a very promising advance in this regard.^286^

### Antimicrobials

Beyond altering genes in the human genome, there are a variety of ways in which genome editing can be used to address human disease and improve human health by targeting the genomes of other organisms. A primary example of this is the recent application of genome editing to attack pathogenic bacterial infections.^24^ For example, gene-editing nucleases can be designed to target genes conferring virulence or antibiotic resistance. Additionally, targeting genome sequences specific to pathogenic strains may facilitate their selective removal from a mixed population. Two recent studies demonstrated proof-of-principle of this approach using the CRISPR/Cas9 system to eliminate bacteria in a mouse skin colonization model^287^ and a moth larvae infection model,^288^ as well as selectively eliminating plasmids and bacterial populations. An intriguing alternative strategy to delivering complete CRISPR systems is to turn native CRISPR systems against themselves by delivery of self-targeted crRNAs, as was recently done to selectively remove bacterial strains with type I CRISPR systems.^289^ The choice of type I systems is notable given that the Cas3 enzyme of type I systems has exonuclease activity that may facilitate DNA disruption and removal,^290^ in contrast to the type II CRISPR/Cas9 system which only cuts DNA. Furthermore, the majority of native CRISPR systems are type I, with only a minor fraction belonging to the type II category.^291^ As with all gene therapies, a primary challenge in moving these platforms forward is the development of a suitable delivery vehicle for clinical translation, and there is promising work in the area of bacteriophage engineering for this purpose.^292^ Collectively, these studies suggest a possible approach to address the rising incidence of antibiotic resistance.

## Conclusion and Future Directions

Tremendous progress has been made in addressing the challenges of conventional gene therapy by developing new technologies for precise modification of the human genome. This has helped to overcome some of the obstacles that have plagued the field of gene therapy for decades. Nevertheless, many challenges still remain to fully realize the potential of genome editing for gene and cell therapy. Central to these challenges are the persistent issues of safety and delivery. In this regard, rapid advances are being made both for increasing the specificity of genome-editing tools and increasing the sensitivity of methods for assessing this specificity genome-wide.^189,190,195,203,204,205,206^ However, it remains unclear whether all off-target effects can be accounted for in a therapy that targets one site within billions of DNA base pairs, involves modification of millions of cells, and is custom prepared for each patient. Moreover, many questions remain about how the human immune system will respond to genetically modified cells or the *in vivo* administration of genome-editing tools. Remarkable advances in delivery technologies are also creating many more opportunities for genome editing, including *ex vivo* delivery to cells with DNA-free components^207,208,209,210,212^ and *in vivo* delivery with efficient and tissue-specific vectors.^220^ The many successes of the preclinical studies reviewed here, as well as the current progression of genome editing in clinical trials, is a source of significant optimism for the future of this field.

The rapid progress in the field is likely to continue to lead to new technologies that will expand the scope of genome editing. Alternative genome-editing technologies, such as targetable site-specific recombinases^293^ that do not rely on the creation of double-strand breaks, alternative CRISPR systems with unique properties,^178,179^ and DNA-guided nuclease systems^294^ will continue to change what is possible with these tools. Epigenome editing, in which DNA-targeting platforms are used to specifically change gene regulation or chromatin structure, is also creating new ways to manipulate the genome for gene and cell therapy.^295,296,297^ Inducible or self-regulating systems that enable the control of the expression, activity, and/or stability of genome-editing tools may play an important role in ensuring their precision and safety. In summary, genome editing has changed the definition of gene and cell therapy and has been a key factor in the recent resurgence of this field, but there is still significant fundamental and translational work to realize the full promise of these technologies for widely treating human disease.

## Figures and Tables

**Table 1:**
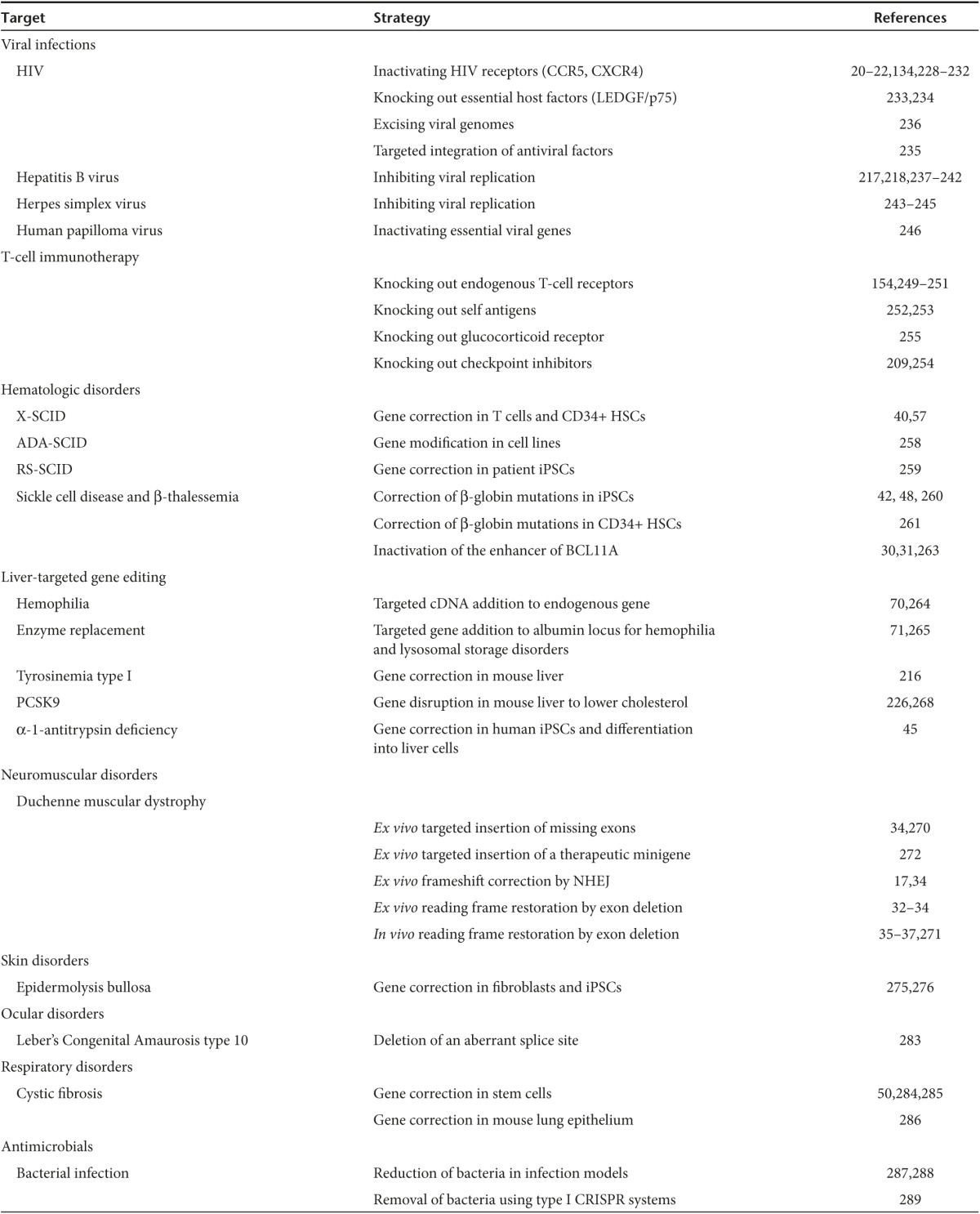
Representative preclinical studies of gene editing for gene and cell therapy

**Table 2:**
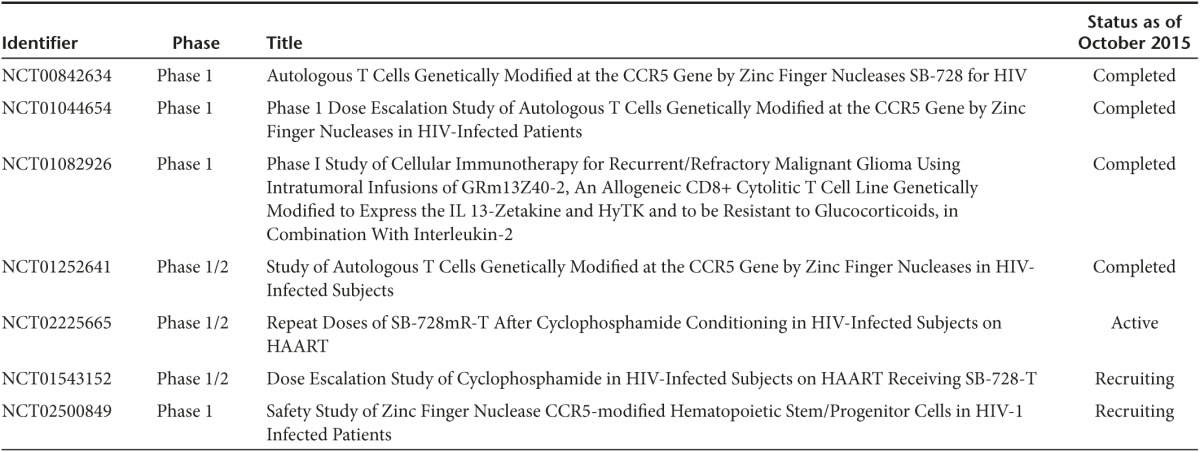
Representative ongoing and completed gene-editing clinical trials
